# 4-1BBL-Armed Oncolytic Herpes Simplex Virus Exerts Antitumor Effects in Pancreatic Ductal Adenocarcinoma

**DOI:** 10.3390/vaccines12121309

**Published:** 2024-11-22

**Authors:** Wenrui Gao, Zhuoqian Zhao, Ying Bi, Jinghua Li, Na Tian, Cuizhu Zhang, Shuyuan Pan, Li Deng, Yuntao Zhang

**Affiliations:** 1 Beijing Institute of Biological Products Company Limited, Beijing 100176, Chinatianna1@sinopharm.com (N.T.); panshuyuan@sinopharm.com (S.P.); 2 College of Life Science, Nankai University, Tianjin 300071, China; 3 China National Biotec Group Company Limited, Beijing 100024, China

**Keywords:** PDAC, oncolytic virus, herpes simplex virus, 4-1BBL, PD-1

## Abstract

**Background:** Pancreatic ductal adenocarcinoma (PDAC) is a highly malignant tumor with a notably poor response to therapy due to its immunosuppressive tumor microenvironment (TME) and intrinsic drug resistance. The oncolytic virus (OV) represents a promising therapeutic strategy capable of transforming the “cold” immunological profile of PDAC tumors to a “hot” one by reshaping the TME. 4-1BB (CD137), a crucial member of the tumor necrosis factor receptor superfamily, plays a significant role in T-cell activation and function. **Methods:** In this study, we constructed an oncolytic herpes simplex virus armed with 4-1BBL (oHSV-4-1BBL), the ligand for the 4-1BB receptor, and investigated its therapeutic effects in two mouse models of pancreatic cancer, Pan02_HVEM and KPC. **Results:** We found that oHSV-4-1BBL remarkably inhibited tumor growth and extended the median survival time in both models. To amplify the therapeutic effect, we further combined oHSV-4-1BBL with PD-1 antibody. This combination therapy not only further suppressed tumor growth but also extended the median survival time by an additional 11 days compared to oHSV (armed with GFP as a control) combined with PD-1 antibody treatment, with some mice achieving complete tumor regression. **Conclusions:** Our findings confirm the potential of combining oncolytic viral therapy with 4-1BB targeting in enhancing the treatment of pancreatic cancer.

## 1. Introduction

Pancreatic cancer, recognized for its high degree of malignancy and grim prognosis, is marked by distressing increases in incidence and mortality rates annually. Most pancreatic cancers are exocrine adenocarcinomas, encompassing pancreatic ductal adenocarcinoma (PDAC) and pancreatic acinar cell carcinoma (PACC). PDAC is the predominant type, accounting for approximately 80% to 90% of all pancreatic cancer cases and being associated with an exceptionally poor prognosis. Conversely, PACC, which represents approximately 1% of pancreatic cancers, has a relatively more favorable prognosis compared to PDAC [1]. Currently, surgical resection remains the most effective treatment option for pancreatic cancer; however, less than 20% of patients are eligible for surgery, with a median survival time of merely 6 months post surgery [2]. Therefore, there is an urgent need to discover innovative and effective treatments.

Oncolytic viruses (OVs), which selectively replicate in and lyse cancer cells, hold significant therapeutic potential for solid tumors and advanced-stage cancers. They not only directly kill cancer cells but also elicit a tumor-specific immune response that aids in the clearance of residual tumors and metastases. Furthermore, when armed with external factors, OVs can perform immune therapeutic functions by expressing immune modulatory factors [3,4].

Many viruses have been employed as oncolytic agents, including adenovirus, reovirus, poxvirus, herpesvirus, rhabdovirus, paramyxovirus, parvovirus, and picornavirus [5,6,7]. Of them, herpes simplex virus type 1 (HSV-1) is the most widely used for OV development [8,9]. HSV-1 is an ideal OV candidate due to its broad cell tropism, robust oncolytic activity, substantial genome space for foreign gene insertion [10], and proven clinical safety record [11,12]. For a better safety profile in cancer treatment, HSV-1 is usually attenuated through genetic modifications such as the deletion of virulence factors [13]. The generation of oncolytic HSV involves the deletion of ICP34.5, which encodes a neurovirulence factor [14,15], as well as ICP47, which enhances MHC I antigen processing and presentation to boost tumor-specific immune responses [16]. Oncolytic HSV has been employed in the treatment of various cancers and has efficiently delivered immune-modulating factors [17,18]. In 2015, talimogene laherparepvec (T-vec; by Amgen, Thousand Oaks, CA, USA) was approved by the FDA for the treatment of advanced melanoma, making it the first and only FDA-approved oncolytic HSV drug to date. It has been investigated in many clinical trials for other types of cancer, including glioma, head and neck cancer, and breast cancer [19].

The genetic manipulation of HSV-1 is essential for its safe and effective use in cancer treatment. Typically, the external genes employed for oncolytic HSV construction are immune modulators, including cytokines, chemokines, checkpoint inhibitors, co-stimulatory ligands, and immune-cell engagers [20]. 4-1BB (CD137, TNFRSF9) is a member of the tumor necrosis factor receptor (TNFR) superfamily, expressed on cell membranes as monomers or dimers, and is a type I transmembrane protein. 4-1BBL (CD137L, TNFSF9) is a ligand for the 4-1BB receptor, which is generally expressed in the form of trimers on the cell membrane and is a type II transmembrane protein [21]. 4-1BBL is predominantly expressed on activated antigen-presenting cells (APCs) such as macrophages, DC cells, and B cells. 4-1BB and 4-1BBL are a pair of costimulatory molecules whose combination provides a second signal for T-cell activation. Many studies have confirmed that 4-1BB and 4-1BBL costimulatory molecules have great application value in cancer treatment [22]. 4-1BBL expressed in APC cells can promote T-cell function and enhance specific antitumor immune responses. Agonistic monoclonal antibodies targeting 4-1BB have been developed for cancer immunotherapy. Preclinical results in various tumor models (both induced and spontaneous) have shown that targeting 4-1BB with agonist antibodies leads to tumor clearance and durable antitumor immunity. Lokon Pharma′s oncolytic virus, LOAd703, is an adenovirus-based oncolytic virus equipped with CD40L and 4-1BBL that has shown potent inhibition of pancreatic cancer in preclinical animal models [23].

To investigate the role of an oncolytic virus in combination with 4-1BB targeting pancreatic cancer treatment, we developed an oncolytic HSV armed with 4-1BBL (oHSV-4-1BBL). In this study, it is demonstrated that oHSV-4-1BBL inhibits tumor growth in both Pan02_HVEM (Pan02 cells expressing herpes virus entry mediator (HVEM)) and KPC pancreatic cancer models. Furthermore, the combination therapy of oHSV-4-1BBL and PD-1 antibody demonstrates enhanced therapeutic efficacy. These findings offer new insights into pancreatic cancer treatment strategies.

## 2. Materials and Methods

### 2.1. Plasmid Construction

The plasmids used to generate oHSV-4-1BBL were constructed as follows. First, the 4-1BBL mouse coding sequence was amplified by PCR from pCMV3-mTNFSF9 (4-1BBL) (Sino Biological, Beijing, China) and inserted into a plasmid with CMV elements. Secondly, the cassette containing the CMV enhancer, CMV promoter, m4-1BBL gene, and SV40 Poly A was cloned into a plasmid with ICP34.5 homology arms.

### 2.2. Cells

African green monkey kidney cell line Vero (originally obtained from ATCC, Manassas, VA, USA); murine pancreatic cell lines MPC-83 (originally from ATCC), KPC (Shanghai Model Organisms Center, Shanghai, China), Pan02 (from Union Hospital, Tongji Medical College, Huazhong University of Science and Technology, Wuhan, China), and Pan02-HVEM (transduced with a lentivirus encoding human HVEM as described previously [24]); and human embryonic kidney cell sublines 293FT and 293FT-sgRNAGFP (stably expressing sgRNA against GFP) were cultured in DMEM (Biological Industries, Goettingen, Germany, 06-1055-57-1ACS) supplemented with 10% fetal bovine serum (FBS, Gibco, Waltham, MA, USA, 16000-044) and 1% penicillin/streptomycin (P/S, Solarbio, Beijing, China, P1400). All cells were incubated at 37 °C with 5% CO_2_.

### 2.3. Virus Construction

The wild-type HSV-1 F strain (wt HSV-1) was obtained from Hongkai Zhang’s laboratory at Nankai University and used for the construction of oncolytic viruses by the CRISPR/Cas9 system as described previously [24,25,26]. Briefly, to generate the ICP34.5-deleted virus, donor DNA plasmids consisting of GFP and homology arms flanking ICP34.5 and sgRNA plasmid targeting ICP34.5 were transfected into 293FT cells. The next day, cells were infected with wt HSV-1. Two days post infection, supernatant containing the produced recombinant virus was serially diluted for plaque purification on Vero cells. The resulting GFP-positive virus was verified by PCR and fluorescence microscopy and further used as the backbone to generate the ICP47 knockout virus. The resulting virus was named oHSV and used as the backbone for further construction. To generate the 4-1BBL-armed oncolytic HSV, donor DNA plasmids containing the 4-1BBL-expressing cassette and homology arms flanking ICP34.5 were transfected into 293FT-sgRNAGFP cells, followed by oHSV infection the next day. Supernatant was harvested for plaque purification and identification of the 4-1BBL-positive virus.

### 2.4. Viral Propagation, Purification, and Concentration

For in vitro analysis, Vero cells were infected with oHSV-4-1BBL at an MOI of 0.05. The supernatant was collected 36 to 48 h after infection. The cell pellets were resuspended with 9% milk and went through three freeze–thaw cycles to release the virus from cells. The virus from the supernatant and the cells were combined, aliquoted and stored at −80 °C. To prepare the purified virus for animal experiments, Vero cells were infected with oHSV-4-1BBL at an MOI of 0.05. The supernatant of the virus culture medium was collected 72 h after infection for centrifugation at 3000 rpm for 10 min, followed by filtration with a 0.45 μm filter (Sartorius, Bohemia, NY, USA, 16533-K). Purification was performed with a HiScreen CaptoCore 700 column (GE Healthcare, Chicago, IL, USA, 17-5481-15) to remove cellular proteins and DNA. The viruses were concentrated with ultrafiltration tubes (Millipore, Burlington, MA, USA, UFC910096).

### 2.5. Plaque Formation Assay

Infectious viral titers were determined with plaque formation assays. Briefly, Vero cells (5 × 10^5^ cells/well) were seeded into 6-well plates. The next day, the culture medium was removed, and serially diluted viruses were inoculated into cells for 2 h at 37 °C, followed by the addition of M199 medium (Biological Industries, 01-080-1ACS) with 1% FBS (Gibco, 16000-044). After 36 to 48 h of incubation at 37 °C with 5% CO_2_, the number of plaques was counted via microscopy for infectious viral titer calculation.

### 2.6. 4-1BBL Expression Analysis by Flow Cytometry

Vero cells, Pan02_HVEM cells, or KPC cells (5 × 10^5^ cells/well) were seeded into 6-well plates. The next day, the cells were infected with oHSV-4-1BBL at an MOI of 1. Two days later, the cells were detached with Accutase (STEMCELL, Vancouver, BC, Canada, 07920), collected by centrifugation, and labeled with fluorescent antibody PE-anti-mouse-4-1BBL (Biolegend, San Diego, CA, USA, 107105). The expression of 4-1BBL was analyzed with BD LSRFortessa X-20.

### 2.7. Cytopathic Effect

Pan02 cells, Pan02_HVEM cells, KPC cells, and MPC-83 were seeded into 6-well plates (5 × 10^5^ cells/well) one day prior to the experiment and infected with viruses at an MOI of 1. The cytopathic effect was observed and photographed 24 h post infection.

### 2.8. Viral Growth Curve

Cells were infected with oHSV or oHSV-4-1BBL at an MOI of 0.01. Then, 0 h, 12 h, 24 h, 36 h, and 48 h after infection, both cells and medium were collected, frozen, and thawed three times. A plaque assay was used to determine the viral titers at each time point.

### 2.9. Cell Viability Assay

Cells were seeded in 96-well plates at a density of 5000 cells per well 6 h prior to infection and infected with serially diluted virus suspension with MOIs of 0.1, 1, and 10. On days 1, 2, and 3 post infection, cell viability was determined by adding 10% cell counting kit 8 (CCK-8) solutions (New Cell & Molecular Biotech, Suzhou, China, C6005-500T). Then, 1–4 h later, optical density (OD) was measured at 450 nm as a readout for cell viability in a microplate reader.

### 2.10. Tumor Therapeutic Model

All animal experiments were conducted in accordance with and approved by the Institute Research Ethics Committee of Nankai University. Six-week-old C57BL/6 mice were purchased from Vital River Laboratories (Beijing, China) and housed under SPF conditions. Mice were subcutaneously injected in the right flank with 5 × 10^5^ viable KPC cells with Matrigel (Corning, Corning, NY, USA, 356237) or 5 × 10^5^ viable Pan02_HVEM cells. When tumors reached approximately 50 mm^3^ (measured with electric calipers and calculated as 1/2 × length × width × width), mice were randomly grouped and intratumorally injected with 5 × 10^6^ plaque-forming units/mouse/100 μL purified oHSV or oHSV-4-1BBL or PBS (100 μL/mouse) as a control. Two more doses were administrated every 3 days after the first injection. For combination treatment, PD-1 antibody was i.p. administered with a dose of 4 mg/kg. The tumor size was measured every 3 days. The tumor size was measured every day as it approached the endpoint of 2000 mm^3^. A mouse was considered dead when the tumor volume reached the endpoint.

### 2.11. Flow Cytometry

In a separate animal experiment, C57BL/6 mice were inoculated with KPC cells and treated as described above, except for that the mice were sacrificed 7 days after the last treatment for intratumoral landscape analysis of immune cells by flow cytometry. Tumors were harvested and digested with collagenase I (Solarbio, D6430), Dispase II (Solarbio, D8071), and Dnase I (Solarbio, R1010) followed by purification with 70 μm restrainer. Red blood cells were lysed with ACK red blood cell lysis buffer (Leagene, Beijing, China CS0001). For staining, cells were first stained with viability dye (BioLegend, 423106) at 4 °C for 10 min. The following antibodies were used: mouse CD45 (BioLegend, 103108), CD 3ε (BioLegend, 100328), CD4 (BioLegend, 100412), CD8a (BioLegend, 100752), CD25 (BioLegend, 102036), CD69 (BioLegend, 104537), Foxp3 (BioLegend, 320014), GZMB (BioLegend, 372208), Ly108 (BioLegend, 134609), LAG-3 (BioLegend, 125226), IFN-γ (BioLegend, 125226), and PD-1 (BioLegend, 135228). Flow cytometry was performed with a BD LSRFortessa X-20.

### 2.12. Statistical Analysis

Statistical analyses were determined by one-way ANOVA using GraphPad Prism 7.0 software, followed by Tukey’s multiple comparison test analysis. All quantitative data are presented as mean ± SD. Mouse survival was plotted using a Kaplan–Meier survival curve and tested for significance with a log rand test. A *p* value less than 0.05 was considered statistically significant (* *p* < 0.05, ** *p* < 0.01,*** *p* < 0.001, **** *p* < 0.0001).

## 3. Results

### 3.1. Construction and In Vitro Characteristics of the oHSV-4-1BBL Virus

**Figure 1:**
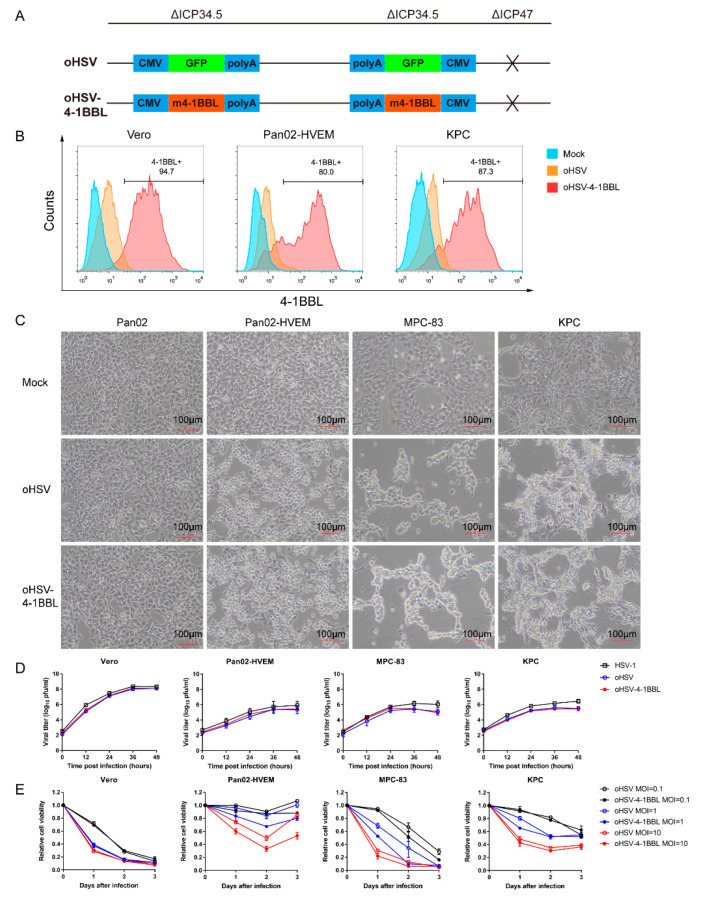
Construction and characterization of the oHSV-4-1BBL virus. (**A**) Schematic diagram of the construction of oHSV-4-1BBL. Two copies of ICP34.5 (neurovirulence factor) were replaced with a gene cassette consisting of the CMV promoter, the GFP or mouse 4-1BBL coding sequence, and SV40 Poly A. In addition, ICP47, which enhances MHC I antigen processing and presentation, was deleted to boost tumor-specific immune responses. (**B**) Expression of 4-1BBL after oHSV-4-1BBL infection in different cells (MOI = 1). After 48 h, the infected cells were collected, blocked with CD16/32 antibody, and stained with PE anti-mouse 4-1BBL antibody, and the expression of 4-1BBL was analyzed by flow cytometry. (**C**) Cytopathic effects caused by viral infection. The cells were infected with the designated virus at an MOI of 1, and an image was taken 24 h post infection. (**D**) Viral replication kinetics. The cells were infected with the indicated virus at an MOI of 0.01, and the titer of the virus at each time point was measured to draw the viral replication kinetics. (**E**) Determination of cell viability after viral infection. Cells were infected with the indicated viruses at three MOIs, and cell viability was monitored at different time points using a CCK-8 kit.

The oHSV-4-1BBL virus used in this study was genetically modified from the existing oHSV virus reported in our previous study [24], which includes the deletion of the ICP34.5 and ICP47 genes and an the insertion of GFP fluorescent protein at the ICP34.5 gene location. To construct the oHSV-4-1BBL virus, the GFP was knocked out using the CRISPR-Cas9 system, followed by homologous recombination of murine 4-1BBL (m4-1BBL) into the location of GFP in the oHSV virus (Figure 1A). ICP34.5 is a neurotoxic factor that, when knocked out, reduces the virus′s resistance to interferon and PKR proteins in normal cells, allowing it to selectively replicate in tumor cells. ICP47 knockout can augment the expression of MHC-I in infected cells, thereby facilitating the presentation of tumor-associated antigens and enhancing the host immune response to tumors. Following viral infection, the loaded 4-1BBL gene can be expressed in tumor cells, binding to the 4-1BB receptor to stimulate T cells to elicit the immune response.

To verify the construction of the virus, a viral genome was extracted and sent out for sequencing. It showed that the 4-1BBL sequence was inserted as designed. In addition, 4-1BBL expression after infection was tested in different tumor cells by flow cytometry. Figure 1B illustrates that 4-1BBL was expressed on the surfaces of Vero, Pan02_HVEM, and KPC cells, indicating the successful construction of the oHSV-4-1BBL.

To assess the impact of the 4-1BBL gene on viral characteristics, we compared the in vitro infection profiles of oHSV and oHSV-4-1BBL in terms of cytopathic effect (CPE), cell viability, and viral replication kinetics. Our findings revealed that both viruses barely induced CPE in Pan02 cells, which lack HSV-1 infection receptors. In contrast, a remarkable CPE was observed post infection with both viruses in Pan02_HVEM, MPC-83, and KPC cells (Figure 1C). This suggests that oHSV and oHSV-4-1BBL share identical infection profiles against pancreatic cancer cells. In terms of the replication kinetics, Vero cells, which are deficient in type I interferon, seemed to be the most susceptible cell line for all three viruses compared to the pancreatic cancer cells. In addition, all three viruses had strikingly similar patterns across all tested cell lines, except that oHSV and oHSV-4-1BBL exhibited slightly lower replication capacity than HSV-1 (Figure 1D). Cell viability was determined to indicate the cytotoxic capacity of oHSV and oHSV-4-1BBL after infection with different MOIs (0.1, 1, and 10). It was demonstrated that the cell viability decreased with time and increases in the MOI for the two viruses. Vero and MPC-83 cells seemed to be more vulnerable than Pan02-HVEM and KPC cells. It should be noted that both oHSV and oHSV-4-1BBL viruses exhibited similar cytotoxic patterns in all cell lines (Figure 1E). Collectively, these results demonstrate that the two viruses have nearly identical characteristics, indicating that the insertion of 4-1BBL did not impact the growth or cytotoxic capacity of the virus.

### 3.2. oHSV-4-1BBL Exhibits Superior Antitumor Activity Compared to oHSV

**Figure 2:**
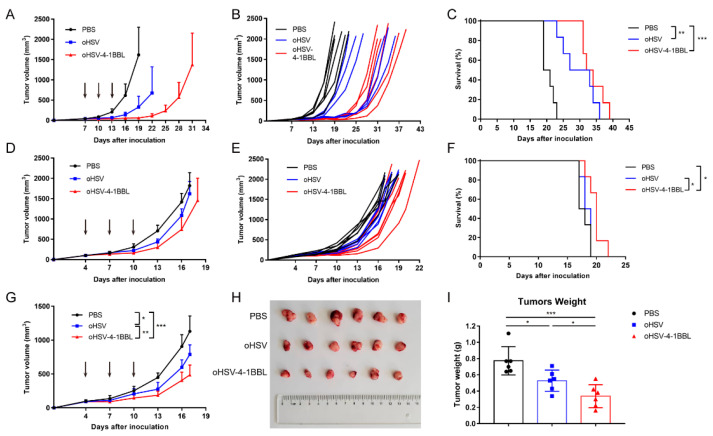
Therapeutic effects of oHSV and oHSV-4-1BBL in pancreatic cancer models. (**A**–**C**) Pan02_HVEM model (*n* = 6). Mice were inoculated with Pan02-HEVM cells on day 0 and treated with PBS, oHSV, or oHSV-4-1BBL on days 7, 10, and 13 (indicated by arrows). Tumor volumes were measured with a caliper every third day. For each group, the average (**A**) and individual (**B**) tumor volume, as well as the survival curve (**C**), are depicted. (**D**–**F**) KPC model (*n* = 6). Mice were inoculated with KPC cells on day 0 and received treatments on days 4, 7, and 10 (indicated by arrows). Tumor volumes were measured with a caliper every third day. For each group, the average (**D**) and individual (**E**) tumor volume, as well as the survival curve (**F**), are documented. (**G**–**I**) KPC model (*n* = 6), which is similar to the experiment demonstrated in (**D**–**E**), except that mice were euthanized on day 17. The tumor growth curves (**G**), tumor images (**H**), and tumor weight (**I**) on day 17 post treatment are illustrated. * *p* < 0.05, ** *p* < 0.01, and *** *p* < 0.001.

To evaluate the antitumor efficacy of the oncolytic virus, we employed two mouse models of pancreatic cancer: Pan02_HVEM and KPC. When tumors reached ~50 mm^3^ (approximately 7 and 4 days post inoculation for Pan02_HVEM and KPC cells, respectively), tumor-bearing mice were treated with PBS or purified oHSV or oHSV-4-1BBL three times (Figure 2A). In the Pan02-HVEM model, both oHSV and oHSV-4-1BBL remarkably inhibited tumor growth compared to the PBS treatment group. It is worth noting that oHSV-4-1BBL showed enhanced antitumor effects relative to oHSV treatment (Figure 2B). For example, on day 22, some mice in the oHSV group had already reached the endpoint, while mice in the oHSV-4-1BBL treatment group were almost cured, despite subsequent recurrence. Similarly, in the KPC model (Figure 2D), which is widely used as a pancreatic cancer model, oHSV-4-1BBL also exhibited better tumor growth inhibition than oHSV (Figure 2E). In terms of survival, both oHSV-4-1BBL and oHSV treatment remarkably extended survival compared to PBS treatment, with oHSV-4-1BBL being associated with the longest survival (Figure 2C,F). For further analysis, we conducted a second experiment with the KPC model. With the same treatment regimen as described above (Figure 2G), mice were euthanized on day 17. Tumor size was significantly reduced in the oHSV-4-1BBL group compared to the PBS and oHSV groups, as evidenced by the visual shape (Figure 2H) and tumor weight (Figure 2I). Taken together, oHSV-4-1BBL exhibited an enhanced antitumor effect relative to oHSV for pancreatic cancers.

### 3.3. Impact of oHSV-4-1BBL Treatment on T Cells

**Figure 3:**
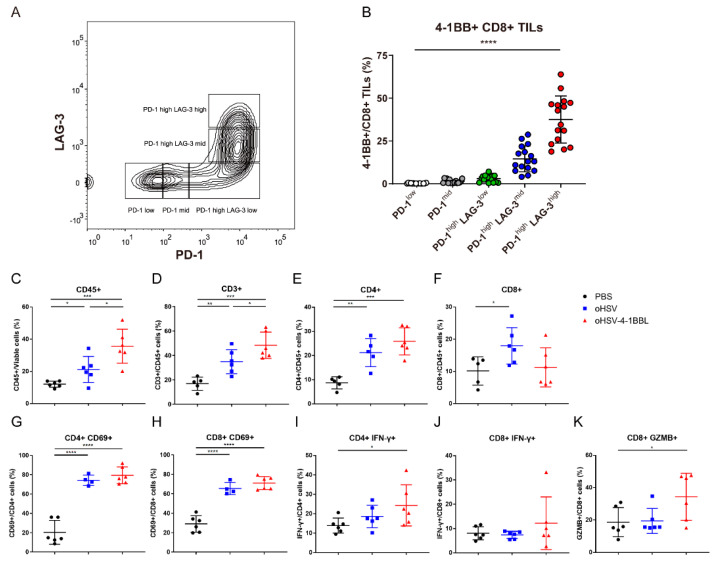
oHSV-4-1BBL treatment promotes T-cell activation and cytotoxicity. (**A**) Classification of KPC tumor model-derived CD8+ TIL cells into distinct exhaustion subsets based on the expression levels of PD-1 and LAG-3. (**B**) Expression of the 4-1BB receptor in different exhausted subsets of CD8+ TIL cells (*n* = 16). oHSV-4-1BBL treatment promotes T-cell infiltration. KPC tumor-bearing mice treated according to the protocol described above were sacrificed on day 17; tumors were isolated and prepared as a single-cell suspension and stained with viability dye and corresponding antibodies. Intratumoral CD45+ cells (**C**), CD45+ CD3+ T cells (**D**), CD45+ CD4+ T cells (**E**), CD45+ CD8+ T cells (**F**), CD69+ CD4+ T cells (**G**), CD69+ CD8+ T cells (**H**), IFN-γ+ CD4+ T cells (**I**), IFN-γ+ CD8+ T cells (**J**), and GZMB+ CD8+ T cells (**K**) were analyzed with flow cytometry. * *p* < 0.05, ** *p* < 0.01, *** *p* < 0.001, and **** *p* < 0.0001.

Given that oHSV-4-1BBL has a better antitumor effect than oHSV, the underlying mechanism remains to be elucidated. Studies have indicated that the 4-1BB receptor is highly expressed in exhausted CD8+ T cells in melanoma and hepatocellular carcinoma [27,28]. To verify whether it is the same case in pancreatic cancer, we examined the expression of the 4-1BB receptor in exhausted CD8+ T cells in the KPC model (Figure 3A,B). CD8+ T cells were categorized into five subgroups based on varying levels of exhaustion markers PD-1 and LAG-3. There was almost no expression of the 4-1BB receptor in the PD-1^low^ and PD-1^mid^ subgroups, with increased expression in the PD-1^high^ LAG-3^mid^ subgroup. As expected, the PD-1^high^ LAG-3^high^ subgroup showed the highest 4-1BB expression. 4-1BBL is mainly expressed by pro-inflammatory antigen-presenting cells, which are rarely found in highly immunosuppressive TMEs, resulting in little stimulation of the 4-1BB receptor. We hypothesized that oHSV-4-1BBL may exert its enhanced antitumor effects by activating T cells and modifying T-cell exhaustion. To verify this hypothesis, the immune landscape of the tumor microenvironment was analyzed. As shown in Figure 3C,D, the number of infiltrated CD45+ and CD3+ cells in tumor tissues in the oHSV-4-1BBL treatment group increased significantly relative to that in the PBS and oHSV treatment groups. oHSV-4-1BBL treatment also boosted the population of CD4+ T cells (Figure 3E) but not that CD8+ T cells (Figure 3F) relative to the other two treatments. Furthermore, activation marker CD69 and cytotoxic markers IFN-γ and granzyme B (GZMB) were analyzed. Compared to the PBS treatment, both oHSV-4-1BBL and oHSV treatments induced significantly larger populations of activated CD4+ and CD8+ T cells (Figure 3G,H), while only the oHSV-4-1BBL treatment resulted in many more IFN-γ-positive CD4+ and CD8+ T cells (Figure 3I,J) and more GZMB+ CD8+ T cells (Figure 3K). Collectively, the above data show that oHSV-4-1BBL treatment promoted the activation and cytotoxicity of T cells in pancreatic tumors.

### 3.4. oHSV-4-1BBL Alters Exhaustive CD8+ T-Cell Distribution

**Figure 4:**
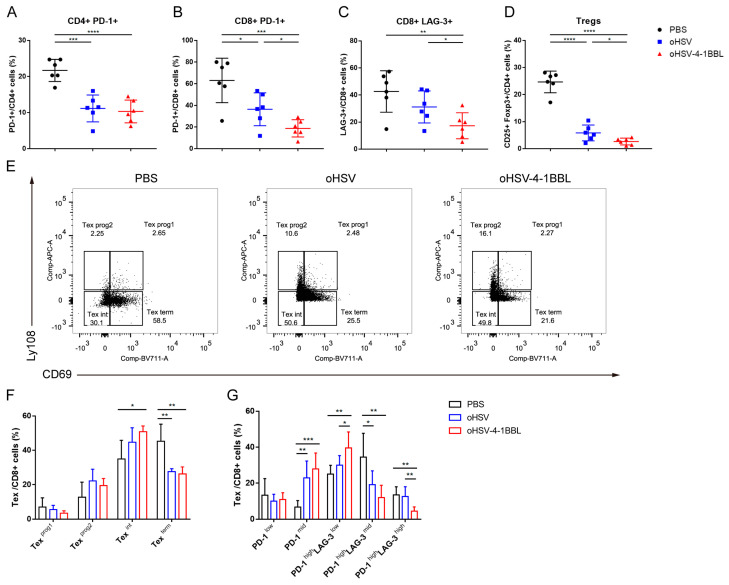
Changes in the distribution of CD8+ T cells at each stage of exhaustion after treatment. (**A**–**D**) oHSV-4-1BBL treatment reduces exhaustive T cells and Treg cells. KPC tumor-bearing mice treated according to the protocol described above were sacrificed on day 17; tumors were isolated, and single-cell suspensions were prepared and stained with corresponding antibodies. CD4+ PD-1+ T cells (**A**), CD8+ PD-1+ T cells (**B**), LAG-3+ CD8+ T cells (**C**), and Treg cells (**D**) were analyzed with flow cytometry. (**E**–**G**) The changes in CD8+ T cells at each stage of exhaustion within the tumor analyzed by two sets of indicators: CD69 and Ly108 (**E**,**F**) and PD-1 and LAG-3 (**G**). * *p* < 0.05, ** *p* < 0.01, *** *p* < 0.001, and **** *p* < 0.0001.

Previously, it was shown that the 4-1BB receptor is highly expressed in exhausted CD8+ T cells in pancreatic tumors (Figure 3B). We further analyzed the population of tumor-infiltrating T cells and regulatory T cells (Tregs). Exhausted CD4+ T and CD8+ T cells were marked by PD-1 and LAG-3, while immunosuppressive Tregs were marked by CD25 and Foxp3. Compared to the PBS treatment, although both OV treatments caused a decrease in exhausted CD4+ T and CD8+ T cells and Tregs (Figure 4A–D), the oHSV-4-1BBL group had a significantly lower population of exhausted CD8+ T cells (Figure 4B,C) and Tregs (Figure 4D) than the oHSV group.

Next, to investigate how exhausted T cells were reduced after oHSV-4-1BBL and oHSV treatments, we examined the distribution of CD8+ T cells at various exhaustion stages in the KPC model after treatment conducted in two different ways. In one approach, based on previous studies [21], CD69 and Ly108 expression levels were used to divide CD8+ T cells into different exhaustion subgroups, as shown in Figure 4E: exhausted progenitor cell 1 (CD69+ Ly108+, Tex^prog1^), exhausted progenitor cell 2 (CD69-ly108+, Tex^prog2^), exhausted intermediates (CD69-ly108-, Tex^int^), and terminally exhausted cells (CD69+ ly108-, Tex^term^). The exhaustion status sequence is Tex^prog1^ → Tex^prog2^ → Tex^int^ → Tex^term^. We found that Tex^prog1^ and Tex^prog2^ had similar trends in the three treatment groups. In the Tex^term^ subgroup, both viral treatment groups showed drastic reductions. However, in the Tex^int^ subgroup, only the oHSV-4-1BBL group showed a significant increase compared to the PBS (Figure 4F).

Another approach was to divide exhausted CD8+ T cells into five subgroups based on high, medium, and low levels of PD-1 and LAG-3 expression (Figure 3A). By comparing the distribution changes of CD8+ T cells at each exhaustion stage under the three treatments, we found that the population of the PD-1^high^ LAG-3^high^ subgroup in the oHSV-4-1BBL treatment group was significantly reduced. In addition, the population of the PD-1^high^ LAG-3^mid^ subgroup was notably decreased in the oHSV-4-1BBL and oHSV treatment groups, but there was no significant difference between them (Figure 4G). In contrast, the two viral treatments markedly increased the population of the PD-1^high^ LAG-3^low^ and PD-1^mid^ subgroups, representing the transition during the exhaustion.

Based on the above analysis that showed similar results, it can be concluded that treatment with oHSV-4-1BBL can dramatically reverse the exhaustion of CD8+ T cells in pancreatic tumors by reducing terminal exhaustion cells and increasing transitional exhaustion T cells.

### 3.5. Combination Therapy with oHSV-4-1BBL and PD-1 Antibody

**Figure 5:**
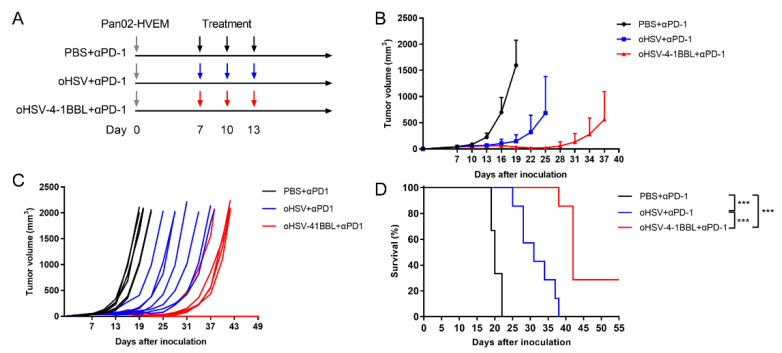
Oncolytic virus combined with PD-1 therapy. (**A**). Regimen of PD-1 antibody combination therapy in the Pan02-HEVM tumor model. (PBS+αPD-1: *n* = 6, oHSV+αPD-1: *n* = 7, oHSV-4-1BBL+αPD-1, *n* = 7). Tumor volumes were measured with a caliper every third day. The tumor growth curves of the average (**B**) and individual mice (**C**) for each group are shown. (**D**) Survival curves of Pan02_HVEM tumor-bearing mice. *** *p* < 0.001.

Despite the fact that oHSV-4-1BBL treatment could reverse the exhaustion of CD8+ T cells (Figure 4F,G) and extended the survival time of tumor-bearing mice, the pancreatic tumors eventually grew without complete regression (Figure 2C,F). Many studies have demonstrated that the combination of immune checkpoint inhibitors contributes to the reversal of CD8+ T-cell exhaustion. We further explored whether blocking the PD-1/PD-L1 signal would further enhance the antitumor effects of the oHSV-4-1BBL treatment. Therefore, a combination therapy of oHSV-4-1BBL and PD-1 antibody was employed in the Pan02-HVEM model. The therapeutic strategy is shown in Figure 5A. PD-1 antibody was injected intraperitoneally at a dose of 4 mg/kg, and simultaneously, oncolytic virus or PBS was administered intratumorally. Tumor growth was monitored post treatment. On day 19, some mice had already reached the endpoint in the PBS+αPD-1 treatment group, with an average tumor volume of 1597.42 mm^3^, while it was only 147.66 mm^3^ and 39.56 mm^3^ for the oHSV+αPD-1 and oHSV-4-1BBL+αPD-1 groups, respectively. On day 25, when some mice in the oHSV+αPD-1 group had reached the endpoint, the mean tumor volume in this group was 684.73 mm^3^, compared to 22.97 mm^3^ in the oHSV-4-1BBL+αPD-1 treatment group (Figure 5B). Throughout the experiment, oHSV-4-1BBL+αPD-1 treatment demonstrated dramatically enhanced inhibition of tumor growth relative to the other two groups, which also significantly extended the survival time of the mice. The median survival was 20, 31, and 42 days for the PBS+αPD-1, oHSV+αPD-1, and oHSV-4-1BBL+αPD-1 groups, respectively. It should be noted that the PD-1 antibody significantly extended survival when combined with oHSV-4-1BBL (42 days vs. 33 days; Figure 2C and Figure 5D), but this was not the case when combined with oHSV (31 days vs. 30 days; Figure 2C and Figure 5D). Notably, two mice were cured in the oHSV-4-1BBL+αPD-1 treatment group. Taken together, it has been shown that the combination of PD-1 antibody could further enhance the antitumor effects of oHSV-4-1BBL compared to the virus alone.

## 4. Discussion

Immunotherapy has made considerable progress in the treatment of tumors, and checkpoint inhibitors such as CTLA-4 and PD-1 have shown some efficacy in non-small cell lung cancer, kidney cancer, and melanoma [29,30] but have shown little efficacy in pancreatic cancer, emphasizing the need for novel therapeutic strategies. The use of oncolytic viruses has shown good therapeutic effects in solid tumors [31,32], and more oncolytic viruses are being developed as antitumor drugs. HSV-1 stands out among them because of its strong oncolytic activity and easy genetic modification.

The superior antitumor activity of oHSV-4-1BBL can be attributed to its dual mechanism of action. First, the oncolytic effect of the virus leads to the destruction of cancer cells and the release of tumor-associated antigens, which can be recognized by the immune system [33]. Secondly, the local expression of 4-1BBL within the tumor microenvironment provides a costimulatory signal to T cells, enhancing their activation and cytotoxic potential.

Considerable studies have shown that T-cell exhaustion in the tumor microenvironment prevents tumors from being destroyed [34]. A typical characteristic of T-cell exhaustion is that cytotoxic capacity is weakened and immunosuppressive receptors are upregulated. Our findings are in line with previous studies that have highlighted the importance of reversing T-cell exhaustion in the tumor microenvironment. The reduction in terminally exhausted CD8+ T cells and the increase in transitional exhausted cells following oHSV-4-1BBL treatment suggest a shift towards a more active T-cell phenotype. This shift is crucial for mounting an effective antitumor immune response.

In this study, we have shown that the efficacy of oHSV-4-1BBL alone in two pancreatic cancer models is limited. This can be attributed to the fact that terminally exhausted CD8+ T cells generally express high levels of the 4-1BB receptor and various immune checkpoint receptors, such as PD-1, LAG-3, Tim-3, and TIGIT. Even if more stimulation of the 4-1BB receptor is performed, it is difficult to alter the suppressive situation. Other studies have found that exciting the 4-1BB signal in T cells increases the secretion of IFN-γ, resulting in the upregulation of PD-L1 in the tumor microenvironment and leading to the re-inhibition of T cells. The combination therapy of oncolytic virus and immune checkpoint inhibitor is a breakthrough in improving the curative effect. Similar to other studies, it was proven in the current study that combination therapy with a PD-1 antibody is beneficial to unlock the great potential of oHSV-4-1BBL for pancreatic cancer treatment. On the contrary, the combination of oHSV and checkpoint inhibitors produced little improvement in efficacy compared to oHSV alone. However, the combination of oHSV-4-1BBL and PD-1 antibody was effective, greatly improving its antitumor effect. Unfortunately, due to some constraints, we were unable to further analyze the tumor-infiltrating T cells after combined treatment with oHSV-4-1BBL and PD-1 antibody.

While our study provides evidence for the efficacy of oHSV-4-1BBL, there are still other aspects to be explored. Future work will focus on how to achieve long-term survival and avoid relapse in mice that achieve complete remission. Additionally, the impact of this combination therapy on other immune-cell populations within the tumor microenvironment will be investigated. Understanding these aspects will be crucial in translating these findings into clinical applications.

## 5. Conclusions

In conclusion, our study has demonstrated promising results in advancing the treatment of pancreatic cancer. The findings underscore the heightened antitumor efficacy of oHSV-4-1BBL, not only by targeting cancer cells directly but also by reversing the exhausted T cells within the tumor microenvironment. Through the innovative application of oHSV-4-1BBL, we have demonstrated that the combination of oncolytic virotherapy with the modulation of the immune checkpoint can elicit a robust and effective antitumor response. Our study provides a foundation for the development of more effective treatments for pancreatic cancer.

## Data Availability

The data presented in this study are available in this article.
